# Heat Stress Affects Faecal Microbial and Metabolic Alterations of Rabbits

**DOI:** 10.3389/fmicb.2021.817615

**Published:** 2022-02-28

**Authors:** Xue Bai, Yu Shi, Lipeng Tang, Li Chen, Huimei Fan, Haoding Wang, Jie Wang, Xianbo Jia, Shiyi Chen, Songjia Lai

**Affiliations:** College of Animal Science and Technology, Sichuan Agricultural University, Chengdu, China

**Keywords:** rabbit, heat stress, metabolome, microbiomics, feces

## Abstract

Heat stress can impair the rabbit immune system, induce oxidative stress, and cause many complications. These diseases are characterized by metabolic disorders, but the underlying mechanism is unknown. As a result, the current research determines the effects of HS on intestinal microorganisms in rabbits and the metabolic pathway disorders caused by HS. Twelve rabbits were randomly assigned to one of two groups: CON (22–24°C) and HS (30°C–32°C). Both the groups were treated for 15 days. Blood and fecal samples were collected on day 15. Serum immune oxidation indices were determined using a commercial ELISA kit, and the microbiome of rabbit feces was studied using 16S rRNA gene sequencing. Non-targeted metabolomics was analyzed using ultra-high-performance liquid chromatography-mass spectrometry (UHPC MS/MS). The findings revealed that HS significantly increased IgG and T-AOC levels in serum, whereas it decreased TNF-α and IL-10. NMDS analysis revealed a substantial difference in bacterial community composition between HS and CON groups. At the phylum level, the abundance of *Firmicutes*, *Protobacteria*, and *Verrucomicrobiota* was significantly higher in the HS group, whereas the abundance of *Bacteriodota* was reduced in the CON group. *V9D2013 group*, *Haloplasma*, *Comamonas*, *Clostridium sensu stricto 1*, *Ruminiclostridium*, *Syntrophus Lutispora*, at the genus level *Syntrophorhabdus*, *Paeniclostridium*, *Clostridium sensu stricto 6*, *Candidatus Caldatribacterium*, *Spirochaeta Synergistaceae*, *Syner-01*, *[Eubacterium] xylanophilum group*, *Cellulosilyticum*, *ADurb.Bin120*, and *Devosia* were significantly upregulated in the HS group. The metabolism of the HS group was considerably upregulated compared with the metabolism of the CON group, according to principal component analysis (PCA) and least-squares discriminant analysis (PLS-DA). HS increased the concentrations of 4-pyridoxic acid, kynurenine, 20-OH-leukotriene B4, and dopamine and decreased the concentration of pyridoxal. In the rabbit gut, these compounds primarily impact the metabolic pathways of vitamin B6, tryptophan, neutrophil activation, and prolactin. 4-Pyridoxic acid, pyridoxal, kynurenine, 20-OH-leukotriene B4, and dopamine are essential inflammatory response markers and oxidative stress.

## Introduction

Heat stress (HS) is a major environmental stress that is detrimental to animal husbandry worldwide (Renaudeau et al., 2012; Sejian et al., 2018). Heat stress is projected to worsen over the next few decades if greenhouse gas emissions continue to rise by the end of the century. The entire globe may be subjected to extreme heat and pressure (Wang and Zhang, 2019). Because of its dense fur, few sweat glands, and sluggish heat dissipation, rabbit, as a thermostatic species, is extremely vulnerable to heat stress (Dalmau et al., 2014). The optimal temperature range for a living environment is between 16 and 21°C (Marai et al., 1994). HS is closely related to the livestock production capacity, which usually leads to the decline of rabbit weight, daily gain, meat quality, and growth rate (Zeferino et al., 2013).

The gastrointestinal tract is governed by a reciprocal circuit comprising the immune and neuroendocrine systems. The intestinal mucosa serves primarily as a barrier between the body’s internal and external environments. Intestinal immunity is one of the primary critical factors in livestock, and it is mainly responsible for growth performance and host health. Research has revealed that HS adversely affects growth, intestinal tissue, feed utilization, and immune health of rabbit (Sirotkin et al., 2021). Metabonomics is a technique for identifying and quantifying all metabolites present in biological samples. At present, Tang et al. (2021) used non-targeted metabonomics to reveal the intestinal pathogenesis and self-healing of rabbits without antibiotic diet, He et al. (2019a) used fecal metabonomics technology to explore the metabolic changes of heat stress in late pregnancy of primiparous sows, and Wen et al. (2021) used metabonomics technology to explore the metabolic changes of heat stress on cecal contents of mice. However, the research on the relationship between heat stress and rabbit intestinal microbial ecosystem and its metabolites is still limited. Therefore, the current paper aims to explore the effects of HS on intestinal microorganisms and metabolism in rabbits, reveal the adverse effects of HS on rabbit health, and understand the mechanism of the adverse effects of heat stress on rabbits.

## Materials and Methods

### Animals and Feeding Strategy

Overall, 48 weaned rabbits (35 days) with similar bodyweight index and health statuses were selected from the Teaching Rabbit Farm of Sichuan Agricultural University. The rabbits were randomly assigned to the control group (CON) and HS group and housed in separate rooms. Briefly, the temperature of the control and HS groups was controlled at 22–24°C and 30–32°C, respectively. The humidity of the two rooms was maintained between 80 and 90%, and the photophase was set to 14L:10D, which lasted for approximately 15 days. Water was freely available; the food was provided twice a day. Each rabbit was kept separately in a clean cage (600 × 600 × 500 mm^3^). The nutritional composition of the rabbits is shown in Table 1. Free feeding was ensured during the experiment. At hours 09:00, 12:00, and 17:00 every day, the anal temperature of each rabbit was measured with an electronic thermometer and recorded.

**TABLE 1:** Nutritional level.

Feed composition	%
Crude protein fifteen	15
Coarse fiber	12.0 ∼ 20.0
Coarse ash eleven	11
Calcium	0.5 ∼ 1.2
Phosphorus	0.4
Sodium chloride	0.3 ∼ 1.0
Water	13.5
Lysine	0.61

*The feed comes from Chengdu Xinjin Jinyang Feed Co., Ltd., meat rabbit (growth) formula feed rp15 Jinyang 550t.*

### Sample Collection and Processing

Six rabbits were randomly selected from the HS and CON groups. The blood and fecal samples were collected after 15 days of the treatment. From 09:00 h, fasting blood samples were collected from the rabbits through the jugular vein puncture. The serum was collected in a 10-ml blood vessel (China Jiangsu Kang Jie Equipment Supply Co., Ltd.) using a gel and clot activator. The blood samples were centrifuged at 3,000× g/min for 15 min at 4°C, and the serum was collected and frozen at −20°C. Then, the rabbits were fixed, their anuses were squeezed, and fresh fecal samples were collected, which were immediately stored in sterile tubes and snap-frozen in liquid nitrogen before storing at −80°C for metabolomic and microbiome analyses.

### Serum Biochemical Parameters Assays

The serum biomarkers of HS, including immunoglobulin G (IgG, DRE-R1952c), interleukin-10 (IL-10, DRE-R0410c), total antioxidant capacity (T-AOC, DRE-R3098c), and tumor necrosis factor-α (TNF-α, DRE-R1360c), were determined using a commercial enzyme-linked immunosorbent assay (ELISA) kit as per the manufacturer instructions (Anya Chengdu Technology Co., Ltd., Chengdu, China). The assay sensitivities were > 0.01 EU/L, 1 ng/ml, 60 ng/L, 100 pg/ml, and 10 pg/ml, respectively.

### 16S rRNA Gene Sequencing

The total genomic DNA of the sample was extracted through the CTAB method, and the DNA concentration and purity were detected on 1% agarose gel. The concentration of each DNA sample was diluted to 1 ng/μl with sterile water. Then, 16S rRNA genes in different regions were amplified using specific primers with a bar code (515F/806R). Then, 15 μl of Phusion was added to the PCR reaction for high-fidelity PCR with the main mixture (New England biological laboratory) using 2 μM forward and reverse primers and approximately 10 ng of the template DNA. The thermal cycle included an initial denaturation at 98°C for 1 min, denaturation at 98°C for 30 times for 10 s, annealing at 50°C for 30 s, extension at 72°C for 30 s, and a final holding at 72°C for 5 min. The same volume of 1X loaded buffer (including the SYBR Green) was mixed with the PCR product and detected by 2% agarose gel electrophoresis.

The PCR products were mixed in an equal density ratio. Then, the Qiagen Gel Extraction Kit (German Qiagen) was used to purify the mixed PCR products. TruSeq was recommended by the manufacturer of the DNA PCR free sample preparation kit (Illumina, United States) to generate the sequencing library and add the index code. The quality of the library was evaluated through Qubit@ 2.0 Fluorometer (Thermo Scientific) and the Agilent Biological analyzer 2,100 system. Finally, the library was sequenced on the Illumina NovasSeq platform, and 250-bp paired-end read codes were generated.

### Metagenomic Data Analyses

According to the barcode and PCR amplified primer sequences, each sample data were separated from the offline data. The barcode and primer sequences were intercepted with the FLASH (V1.2.7).^1^ The reads of each sample were spliced, and the spliced sequences were the original data tags (raw tags). We referred to the Qiime (V1.9.1)^2^ process to obtain a high-quality tag sequence. These tags sequences were passed.^3^ The species’ annotation databases were compared to detect the chimera sequence. The chimera sequences were finally removed to obtain the final adequate data.

Using the uparse algorithm (uparse v7.0.1001),^4^ all the effective tags of samples were clustered. By default, the sequences were clustered into the operational taxonomic units (OTUs) with 97% identity. Meanwhile, the sequence with the highest frequency in OTUs was selected as the representative sequence of OTUs. The music (version 3.8.31)^5^ software was employed to perform fast multi-sequence alignment to determine the phylogenetic relationship of OTU representative sequences. Finally, the data of each sample were homogenized.

### Fecal Metabolomic Profiling

Non-target metabolomics analyses were performed at Novogen Co., Ltd. (Beijing, China) using ultra-high-performance liquid chromatography combined with mass spectrometry (UHPLC-MS/MS) and the vanquish UHPLC system (Thermo Fisher Scientific, Germany) and Orbitrap Q ACTIVETM HF mass spectrometer (Thermo Fisher Scientific).

### Metabolite Recognition

The raw data file generated by the UHPLC-MS/MS was processed using compound discoverer 3.1 (CD3.1, Thermo Fisher Scientific) to perform peak alignment, peak pickup, and quantification for each metabolite. The primary parameter settings were as follows: retention time tolerance, 0.2 min; actual quality tolerance, 5 ppm; signal strength tolerance, 30%; and signal-to-noise ratio, 3. Then, the peak intensity was normalized to the total spectral intensity. The normalized data were applied to predict the molecular formula based on additive ions, molecular ion peaks, and fragment ions. Then, the peak was matched to mzCloud.^6^ Statistical software R (R version r-3.4.3), python (Python version 2.7.6), and CentOS (CentOS version 6.6) were applied for statistical analyses. We used the area normalization method for normal transformation when the data were not normally distributed.

### Correlational Analyses Between Intestinal Microorganisms and Specific Metabolites

Pearson’s statistical analysis was used to analyze the correlation at the genus level between the differential metabolites of the top 20 (sorted from small to large according to the *p*-value) and the differential bacterial genera of the top 10 (sorted from small-to-large according to the *p*-value). The relative abundance of each differential bacterial genus level and the *p*-value between different differential metabolites were calculated.

### Statistical Analyses

SPSS 25.0 software (IBM, Chicago, Illinois, United States) was employed for statistical analyses. The differences between the two groups were compared through a *t*-test. For similarity analysis of multivariate data (Anosim), we adopted the R software^7^ for comparing the bacterial community structure. For serum analysis, the data were subjected to a *t*-test using the Graphpad Prism 6 software (San Diego, CA, United States). *p* < 0.05 was considered statistically significant.

## Results

### Anal Temperature and Serum Parameters

**FIGURE 1:**
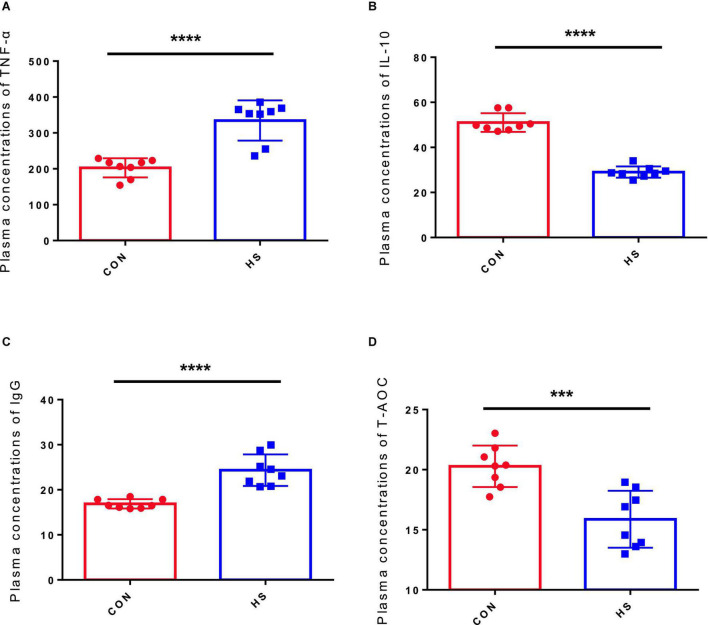
Effect of HS on the serum hormone levels in rabbits. **(A)** IgG concentration in the serum; **(B)** IL-10 concentration in the serum; **(C)** TNF-α concentration in the serum; **(D)** T-AOC concentration in the serum. The asterisk symbols indicate significance (***) and more significant (*****).

Table 2 displays the statistical data of anal temperature. Then, to assess the potential effects of HS on the body, we discussed the effects of HS on oxidative stress and immune response. The TNF-α, T-AOC, IL-10, and IgG levels in the CON and HS groups were measured using serum samples. TNF-α (Figure 1A) and IgG (Figure 1C) levels in the HS group increased significantly (*p* < 0.05) compared with the CON group, although IL-10 (Figure 1B) and T-AOC (Figure 1D) levels decreased significantly (*p* < 0.05).

**TABLE 2:** Anal temperature.

HS group	CON group
38.61°C	37.43°C

*Mean Anal temperature of 12 randomly selected rabbits three times a day during treatment.*

### Fecal Microbial Community

**FIGURE 2:**
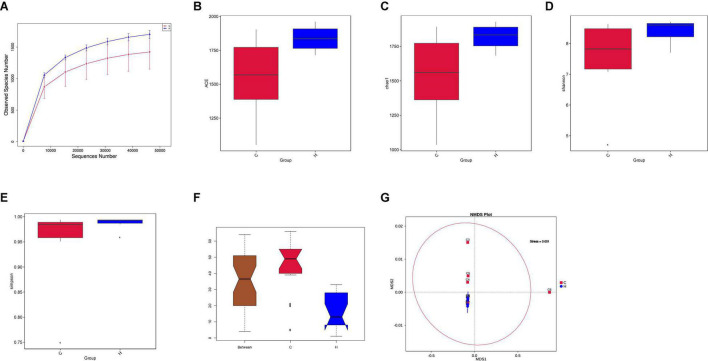
Effects of HS on the fecal microbiota of rabbits. **(A)** Rarefaction curves (the mean curves for the samples/group) were used to plot the number of phylotypes detected in the 16S rDNA gene libraries by the number of sequences from the fecal microbiota of rabbits in the HS and CON groups. **(B)** Box chart of ACE index difference between the HS and CON groups. **(C)** Box chart of Chao index difference between the HS and CON groups. **(D)** Box chart of Shannon index difference between the HS and CON groups. **(E)** Box chart of Simpson index difference between the HS and CON groups. **(F)** Rank significance test of the fecal community between the HS and CON groups based on the Bray–Curtis distance metric (ANOSIM analysis). *R*-value > 0, the difference between the groups was significant **(G)**. Non-Metric Multi-Dimensional Scaling (NMDS) ordination plots of the fecal bacterial communities in the HS and CON groups based on the Bray–Curtis distance metric. HS, heat stress; CON, control.

**FIGURE 3:**
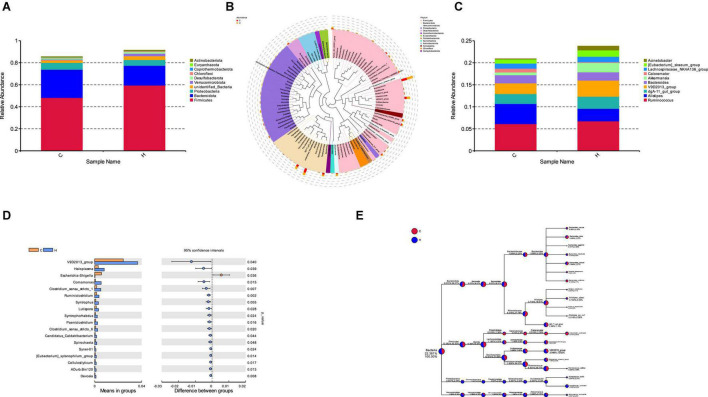
Effect of HS on the rabbit fecal microorganisms. **(A)** Histogram of species relative abundance at the phylum level; **(B)** phylogenetic relationship of the species at the genus level. **(C)** Histogram of the species’ relative abundance at the genus level; **(D)** different species at the genus level in the HS and CON groups. **(E)** Dendrogram depicting the flora expression.

Fresh fecal samples were obtained from 6 HS rabbits and 6 CON rabbits after 15 days of the treatment to investigate the effect of HS on intestinal microbiota composition. Averages of 92,858 labels were assessed in each of the 12 samples, and the effective quality control rate was 66.61% (Table 3). The sparse curve (the average curve of each group) demonstrated that most microbial diversity had been fully recorded (Figure 2A). According to the Wilcoxon rank-sum test, no significant difference in ACE (Figure 2B), Chao (Figure 2C), Shannon (Figure 2D), or Simpson indices (Figure 2E) between the HS and CON groups was observed (*P* > 0.05). However, some substantial differences in microbial composition between the two groups were observed due to HS. According to anosim analysis, the difference between groups was bigger than the difference between groups (*R*-value = 0.1611 > 0, *p* < 0.05) (Figure 2F). The NMDS ranking diagram, based on Bray Curtis distance measurement, demonstrated that the fecal bacterial population in the sample was separated by HS (Figure 2G). With 97% identity, sequence aggregation combines OTU (operational taxon). The silva138 database annotated the OTU sequences after obtaining 3,392 OTUs. When the silva138 database, species annotation, and statistical data from various classification levels were compared, we discovered that, of 3,392 OTUs, 3,386 (99.82%) could be annotated to the database and 84.40% to the gate level. Taxonomic component analysis with a relative abundance of more than 1% was performed to define the bacterial taxa induced by HS. The rabbit fecal bacterial population was dominated by two major phyla in the CON group (Figure 3A). *Firmicutes* were the most abundant, with an average abundance of 48.20%, followed by *Bacteriodota* (25.65%). In the HS group, *Firmicutes* (59.51%) also occupied an absolute advantage, followed by *Bacteriodota* (17.93%), *Protobacteria* (5.23%), and *Verrucomicrobiota* (2.33%). In the fecal microbiota of rabbits in the HS group, the abundance of *Firmicutes*, *Proteobacteria*, and *Verrucomicrobiota* tended to increase, whereas the abundance of *Bacteriodota* decreased. To investigate the evolutionary relationships of species at the genus level, sample sequences from the Top100 genera were acquired through multiple sequence alignment, as shown in Figure 3B. *Ruminococcus (Firmicutes*) were the most abundant genus of rabbit intestine microbes at the genus level, and HS would increase the quantity of *Firmicutes.* Similarly, the HS group exhibited higher *Akkermansia* (*Verrucomicrobiota*) and *Acinetobacter* (*Proteobacteria*). In contrast, *Alistipes (Bacteroidota)* decreased in the HS group (Figure 3C). As shown in Figure 3D, the *t*-test between the groups revealed that, compared with CON group, in the HS group, *V9D2013 group*, *Haloplasma*, *Comamonas*, *Clostridium sensu stricto 1*, *Ruminiclostridium*, *Syntrophus Lutispora*, *Syntrophorhabdus*, *Paeniclostridium*, *Clostridium sensu stricto 6*, *Candidatus Caldatribacterium*, *Spirochaeta Synergistaceae;g Syner-01*, *[Eubacterium] xylanophilum group*, *Cellulosilyticum*, *ADurb.Bin120*, and *Devosia* were significantly upregulated, whereas *Escherichia Shigella* was significantly downregulated (*p* < 0.05). In addition, as shown in Figure 3E, we drew the expression tree of the most abundant flora in the realm, phylum, class, order, family, genus, and species.

**TABLE 3:** Data preprocessing, statistics, and quality control.

Sample name	Raw PE	Effective%
H1	103,787	63.16
H2	100,539	66.88
H3	76,203	70.15
H4	70,660	70.57
H5	91,939	70.61
H6	91,860	68.67
C1	92,541	67.00
C2	90,500	67.06
C3	84,512	75.91
C4	103,272	60.56
C5	106,091	59.77
C6	102,391	58.98
Average	92857.92	66.61

**FIGURE 4:**
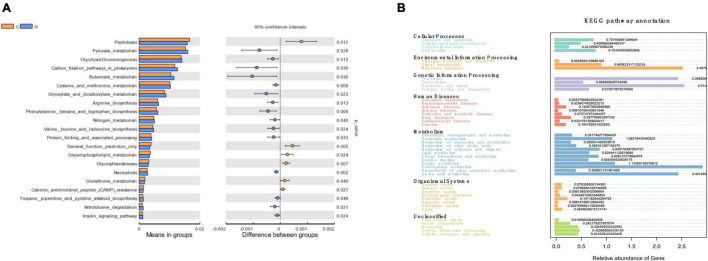
The KEGG pathway affected by HS in rabbits. **(A)** The KEGG levels 1 and 2; **(B)** KEGG level 3.

These bacteria were subjected to a KEGG analysis, which revealed substantial differences. The six most prevalent metabolic pathways in the KEGG Level 2 (Figure 4A) of HS microbiota were carbohydrate metabolism, amino acid metabolism, energy metabolism, nucleoside metabolism, cofactor and vitamin metabolism, and glycan biosynthesis and metabolism. Figure 4B depicts the 21 most prevalent KEGG pathways in the third tier of the KEGG hierarchy. Peptidase, pyruvate metabolism, glycolysis/gluconeogenesis, the prokaryotic carbon fixation pathway, butancate metabolism, cysteine and methionine, glyoxylate, and dicarboxylate were the most prevalent. Considerable disparities between the HS and CON groups might be observed, demonstrating that HS significantly influenced several pathways. Necroptosis, phenylalanine, tyrosine, tryptophan biosynthesis, cysteine and methionine metabolism, valine, leucine, and isoleucine biosynthesis, arginine biosynthesis, glyoxylate, and dicarboxylate metabolism, pyruvate metabolism, butanoate metabolism glycolysis/gluconeogenesis insulin signaling pathway increased significantly in the HS group. However, in the HS group, the inositol phosphate metabolism, glycerophospholipid metabolism, and phosphatidylinositol signaling system pathways were dramatically reduced (Table 4). These markedly changed pathways were associated with metabolism (primary KEGG function).

**TABLE 4:** KEGG pathway affected by heat stress in rabbits.

KEGG level 1	KEGG level 2	KEGG level 3	avg(C)	sd(C)	avg(H)	sd(H)	*p*-value
Cellular processes	Cell growth and death	Necroptosis	0.002681	5.36E-05	0.002821	6.39E-05	0.002261
Metabolism	Amino acid metabolism	Phenylalanine tyrosine and tryptophan biosynthesis	0.005733	0.00027	0.006177	0.000174	0.008745
		Cysteine and methionine metabolism	0.010162	8.98E-05	0.010323	8.33E-05	0.009308
		Valine, leucine, and isoleucine biosynthesis	0.004549	0.00019	0.004796	0.000101	0.023774
		Arginine biosynthesis	0.006115	0.000184	0.006387	7.18E-05	0.013368
	Carbohydrate metabolism	Glyoxylate and dicarboxylate metabolism	0.008336	0.000391	0.008848	0.000213	0.02341
		Pyruvate metabolism	0.015284	0.000615	0.016052	0.0003	0.027544
		Butanoate metabolism	0.010459	0.000827	0.011488	0.000498	0.030375
		Glycolysis/Gluconeogenesis	0.01384	0.000173	0.014108	0.000119	0.012393
		Inositol phosphate metabolism	0.000737	8.65E-05	0.000643	3.27E-05	0.044643
	Lipid metabolism	Glycerophospholipid metabolism	0.003698	0.000206	0.003433	7.12E-05	0.024122
Environmental information processing	Signal transduction	Phosphatidylinositol signaling system	0.000384	2.22E-05	0.000355	1.33E-05	0.023136
Organismal systems	Endocrine system	Insulin signaling pathway	0.001154	7.19E-05	0.001262	6.92E-05	0.024403

### Fecal Metabolite Profiles

**FIGURE 5:**
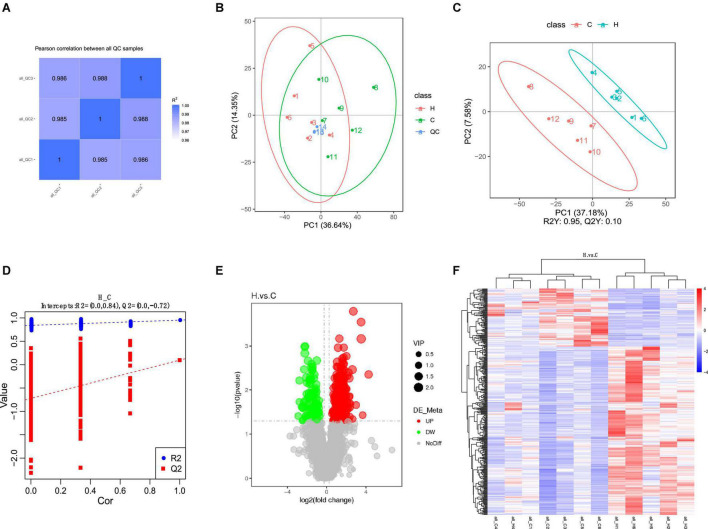
Effect of HS on fecal metabolome of rabbits. **(A)** QC sample correlational analysis chart; **(B)** PCA value of the main sample; **(C)** PCA value of the principal component; **(D)** PLS-DA; **(E)** differential metabolite volcano map, the abscissa represents the difference multiple change (log_2_-fold change) of metabolites in different groups, and the ordinate represents the difference significance level (-log_10_ *p*-value). Each point in the volcanic map represents a metabolite, the significantly upregulated metabolites are represented by red dots, the significantly downregulated metabolites are represented by green dots, and the size of the dot represents the VIP value; **(F)** cluster analysis of differential metabolites.

The correlation analysis chart of QC samples (Figure 5A) demonstrates that the detection method is steady and excellent data quality. The PCA values of the main samples (Figure 5B), PCA values of the main components (Figure 5C), and PLS-DA findings (Figure 5D) revealed variations in intestinal metabolism between the CON and HS groups. The model quality metrics are *R*2 = 0.84 and *Q*2 = 0.72 for positive model data. These findings demonstrate that the PLS-DA model is accurate and does not overfit. The HS vs. CON comparison identified 1,545 metabolites, including 382 differential metabolites (*p* < 0.05), 284 significantly upregulated metabolites, and 98 significantly downregulated metabolites (Tables 5, 6 differential metabolites, *p* < 0.01), and the water levels of these 382 metabolites are shown in the volcanic map (Figure 5E) and heat map (Figure 5F).

**TABLE 5:** Metabolite difference screening results.

Compared samples	Num. of total ident.	Num. of total sig.	Num. of sig. up	Num. of sig. down
H.vs. C all	1,545	382	284	98

**TABLE 6:** Differential metabolites (*p* < 0.01).

Name	Formula	*P*-value	AUC	VIP	Up. Down
4-Pyridoxic acid	C6 H8 N2 O3	0.000164291	1	1.724335224	Up
N-(4-chlorophenyl)-N’-cyclohexylthiourea	C15 H18 O5	0.000683365	1	1.515194843	Up
N-METHYL (-) EPHEDRINE	C11 H15 N2 O8 P	0.000689312	1	2.107021897	Up
(5S)-5-hydroxy-1,7-diphenylheptan-3-one	C10 H20 O7 P2	0.001089915	1	1.457631076	Up
(6E)-7-(2H-1,3-benzodioxol-5-yl)-1-(piperidin-1-yl) hept-6-en-1-one	C5 H13 O14 P3	0.001095926	1	1.627533423	Up
Sarpogrelate	C6 H8 N2 O3	0.001713463	1	1.499162298	Up
δ-Gluconic acid δ-lactone	C13 H19 N5 S	0.001828088	1	1.43668192	Up
20-Carboxy-Leukotriene B4	C12 H18 N2 O2	0.001923798	1	1.443357407	Up
9-KODE	C11 H13 N O3	0.00224591	1	1.427332021	Up
1,7-bis(4-hydroxyphenyl) heptan-3-one	C9 H8 N2 O2	0.002298406	1	1.602919688	Up
Chelidamic acid hydrate	C22 H23 N O4	0.002509887	1	1.464795628	Up
penta-1,4-dien-3-one	C17 H19 N5	0.002869227	1	1.525811465	Up
MGDG (12:0/16:0)	C16 H17 N O8	0.003515374	1	1.363611352	Up
Asp-glu	C26 H31 N O4	0.005065832	1	1.395985074	Up
Isoferulic acid(IFA)	C9 H14 N5 O4 P	0.005127715	1	1.331357281	Up
3-pentadecyl-4,5,6,7-tetrahydrobenzo[d]isoxazol-4-one oxime	C17 H25 N O4	0.005340553	1	1.388075195	Up
Cryptotanshinone	C8 H15 N3 O4	0.005704609	1	1.331796664	Up
4-(3,4-dihydro-2H-1,5-benzodioxepin-7-ylamino)-4-oxobutanoic acid	C19 H17 N3 S	0.009267392	1	1.394537236	Up
2-Methoxyestradiol	C48 H78 O18	0.001468525	1	1.737971167	Down
D-Mannitol 1-phosphate	C10 H18 N2 O5 S	0.002175742	1	1.675900171	Down
FAHFA (2:0/18:1)	C29 H44 O12	0.004452236	1	1.742404251	Down
Pyridoxine	C24 H49 O9 P	0.005178639	1	1.590221982	Down
13,14-Dihydro-15-keto-tetranor prostaglandin F1α	C21 H20 O6	0.005888753	1	1.46518548	Down
4-(1,2,3-thiadiazol-4-yl) phenyl pivalate	C15 H10 O4	0.006092308	1	1.376454299	Down
L-Anserine (beta-alanyl-N-methylhistidine) (nitrate salt)	C18 H22 O2	0.006735438	1	1.580915809	Down
8-iso-15-keto Prostaglandin F2α	C14 H16 N4 O S	0.000288108	0.972222222	1.71227909	Up
N1-(2,4-difluorophenyl)-2-morpholino-2-thioxoacetamide	C18 H27 N O4	0.002992593	0.972222222	1.391777974	Up
Homocysteic acid	C10 H16 N2 O3 S	0.003303585	0.972222222	1.434996298	Up
T-2 Triol	C9 H10 O5	0.003307428	0.972222222	1.414491412	Up
1-Caffeoylquinic Acid	C9 H10 Cl N O3	0.003449961	0.972222222	1.82737873	Up
L-Hydroxylysine	C6 H9 N O S	0.004439426	0.972222222	1.594098636	Up
L-Kynurenine	C11 H12 N2 O4	0.004610227	0.972222222	1.344000995	Up
AB-PINACA N-(2-fluoropentyl) isomer	C22 H25 N O6	0.004798916	0.972222222	1.345094803	Up
Desthiobiotin	C12 H18 O7	0.005121097	0.972222222	1.465348583	Up
VPH	C18 H20 Cl N3 O	0.005418981	0.972222222	1.338432359	Up
6,7-Dihydroxycoumarin	C20 H24 O2	0.005843015	0.972222222	1.335174432	Up
Thromboxane B2	C14 H25 N5 O7	0.00691914	0.972222222	1.370510321	Up
N-Acetyl-L-histidine	C16 H22 N2 O3	0.007042218	0.972222222	1.311288166	Up
Thymine	C10 H19 N O3	0.007098831	0.972222222	1.287747789	Up
Noroxymorphone	C17 H18 N2 O5	0.007222451	0.972222222	1.420811053	Up
L-Ascorbic acid 2-sulfate	C11 H17 N O3	0.009874913	0.972222222	1.277309559	Up
Nicotinamide	C15 H19 N O2	0.009987374	0.972222222	1.294591044	Up
N-Acetyl-L-glutamic acid	C6 H11 N O4	0.002959442	0.944444444	1.384600905	Up
HNH	C24 H33 N3 O4	0.004486986	0.944444444	1.362712099	Up
2-({2-oxo-2-[(2-oxo-3-azepanyl) amino]ethyl}sulfanyl) acetic acid	C8 H11 N O3	0.00466919	0.944444444	1.336762578	Up
GPK	C8 H9 N O2	0.005315694	0.944444444	1.319334025	Up
FRH	C11 H9 N O3	0.005364114	0.944444444	1.795879214	Up
2-Hydroxy-2-methyl-3-buten-1-yl beta-D-glucopyranoside	C13 H16 N2 O3	0.005388991	0.944444444	1.426111495	Up
N1-[(1-phenyl-1H-pyrazol-3-yl) methylidene]-4-chloroaniline	C20 H32 O5	0.005458972	0.944444444	1.444143628	Up
NNK	C16 H18 F N3 O3	0.005884176	0.944444444	1.307892114	Up
O-7460	C11 H11 N3 O2	0.00681632	0.944444444	1.464662074	Up
Valylproline	C14 H19 N O3	0.007895287	0.944444444	1.321901061	Up
LPE 14:0	C10 H14 N2 O4	0.008077976	0.944444444	1.3059036	Up
Lysope 18:1	C15 H13 N3 O	0.008428624	0.944444444	1.282964775	Up
17alpha-Ethinyl estradiol	C15 H13 N O2 S	0.008483312	0.944444444	1.312680927	Up
Choline Glycerophosphate	C16 H14 N2 O	0.008736751	0.944444444	1.296395901	Up
4-Hydroxy-2-Oxoglutaric Acid	C5 H9 N O3 S	0.009017021	0.944444444	1.289984482	Up
2-{[methyl (2,3,4,5,6-pentahydroxyhexyl)amino]methylidene}malononitrile	C17 H19 N3 O3 S	0.009489953	0.944444444	1.298949287	Up

The area under the curve (AUC) was used to assess the sensitivity and specificity of biomarkers in predicting the occurrence of events. The major HS metabolites were 4-pyridoxic acid, N-(4-chlorophenyl)-N-cyclohexylthiourea, N-METHYL (-)EPHEDRINE, (5S)-5-hydroxy-1,7-diphenylheptan-3-one, (6E)-7-(2H-1,3-benzodioxol-5-yl)-1-(piperidin-1-yl) hept-6-en-1-one, sarpogrelate, δ-gluconic acid δ-lactone, 20-carboxy-leukotriene B4, 9-KODE, 1,7-bis (4-hydroxyphenyl) heptan-3-one, chelidamic acid hydrate, penta-1,4-dien-3-one, MGDG (12:0/16:0), Asp-Glu, isoferulic acid (IFA), 3-pentadecyl-4,5,6,7-tetrahydrobenzo[d]isoxazol-4-one oxime, cryptotanshinone, 4-(3,4-dihydro-2H-1,5-benzodioxepin-7-ylamino)-4-oxobutanoic acid, 2-methoxyestradiol, D-mannitol 1-phosphate, FAHFA (2:0/18:1), pyridoxine, 13,14-dihydro-15-keto-tetranor prostaglandin F1α, 4-(1,2,3-thiadiazol-4-yl) phenyl pivalate, and L-anserine (beta-alanyl-N-methylhistidine) (nitrate salt) (AUC = 1).

**FIGURE 6:**
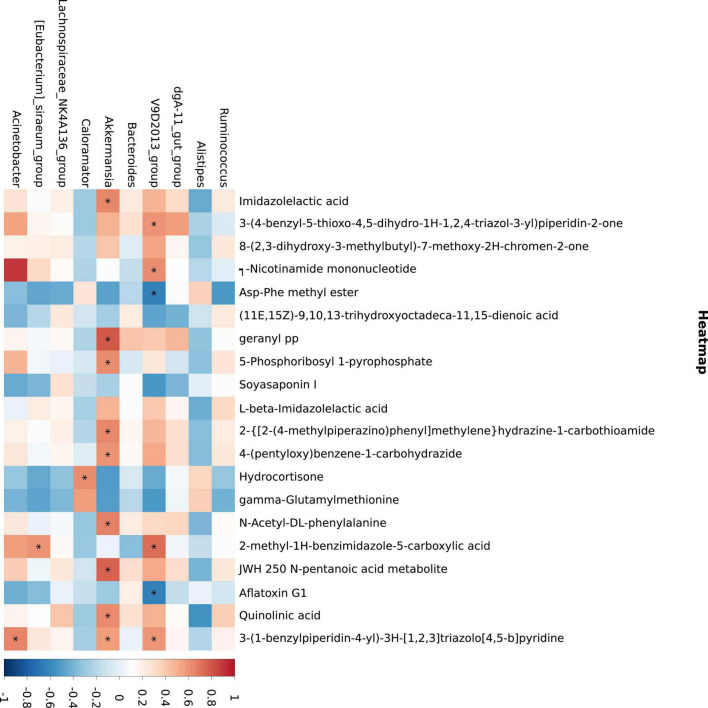
Metabolite and microbial association analyses. The abscissa is the differential metabolite (top 20) and the ordinate is the 16S differential bacteria (top 10). Blue indicates negative correlation, while red indicates positive correlation. *p ≤ 0.05.

The results of differential metabolites correlation analysis (Figure 6) revealed a strong positive correlation between imidazolectic acid and 2-{[2-(4-methylpiperazino) phenyl] methyl} hydrazine-1-carbohydramide and 4-(pentyloxy) benzene-1-carbohydrazide (Pearson correlation coefficient > 0.95). 3-(1-Benzylpiperidin-4-yl)-3H-[1,2,3] triazolo [4,5-b] pyridine was negatively correlated with ASP Phe methyl ester and gamma glutamylmethionine, but positively correlated with jwh 250 n-pentanoic acid metabolite and quinolinic acid (Pearson correlation coefficient < −0.75) (Table 6).

**FIGURE 7:**
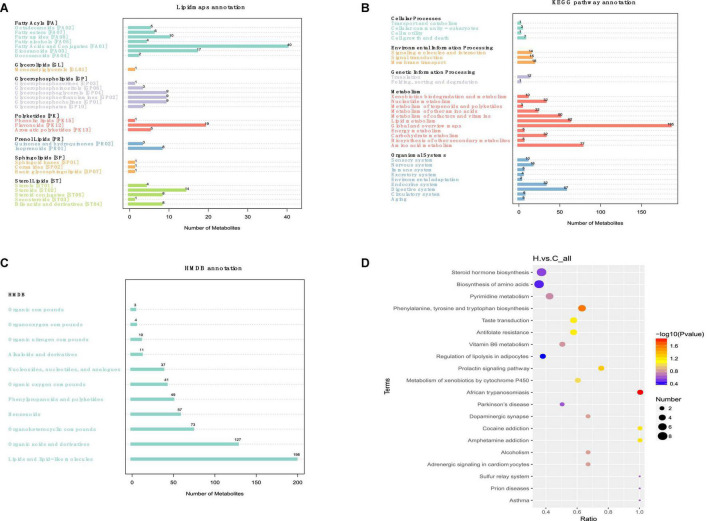
Effects of HS on the metabolic pathway and metabolism of rabbit fecal metabolites. **(A)** Lipid maps’ classification notes; **(B)** KEGG access notes; **(C)** HMDB classification notes; **(D)** the KEGG Enrichment Bubble Diagram.

The principal KEGG annotation pathway findings revealed that distinct metabolites were mostly engaged in global and overview maps, amino acid, lipid, cofactors and vitamins, carbohydrate, nucleoside, and other metabolic pathways. The primary biological roles of various metabolites were determined using KEGG (Kyoto Encyclopedia of genes) pathway enrichment analysis. They are associated with biological activities, including cell development and death. Furthermore, it is related to organic systems such as digestive, endocrine, neurological, sensor, immunological, and circulatory (Figure 7A). The human metabolome database (HMDB) secondary classification annotation results show that metabolites are primarily involved in lipids and Lipids and lipid-like molecules, Organic acids and derivatives, Organoheterocyclic compounds, Benzenoids, Phenylpropanoids and polyketides, Organic oxygen compounds, and other pathways (Figure 7B). Table of categorization annotation results for LIPID MAPS: metabolites in Ming Dynasty include mostly Fatty Acids and Conjugates [FA01], Flavonoids [PK12], Eicosanoids [FA03], Steroids [ST02], Fatty amides [FA08], Glycerophosphocholines [GP01], Glycerophosphoethanolamines [GP02], and Glycerophosphoglycerols [GP04] lipid classification (Figure 7C). According to the KEGG data, HS influenced the rabbit metabolic pathway, and linked endogenous chemicals were most abundant in tryptophan biosynthesis, VB6 metabolism, the prolactin signaling pathway, and xenobiotic metabolism *via* the cytochrome P450 metabolic pathway (Figure 7D).

### Correlation Analysis Between Intestinal Microorganisms and Specific Metabolites

*Akkermansia* has a strong positive correlation with a variety of metabolites, mainly involved in the metabolism of imidazolectic acid, geranyl PP, JWH 250 n-pentanoic acid metabolite, quinolinic acid, and 3-(1-benzylpiperidin-4-yl)-3H-[1,2,3] triazolo [4,5-b] pyridine. Figure 6 depicts the findings of the correlation. It should be noted that *V9D2013 Group* was inversely related to Asp-Phe methyl ester and aflatoxin G1.

## Discussion

We revealed that HS significantly affects rabbit intestinal microbiota (microbial composition and metabolism). In the last 2 years, studies on the impact of HS on microbial composition in broilers, laying hens, ducks, goats, and dairy cows have been published (He et al., 2019b; Zhong et al., 2019; Zhu et al., 2019; Li et al., 2020; Rostagno, 2020). However, the influence of HS on the microbial composition and metabolites of rabbits is yet unclear. As a thermostatic animal, rabbits in an ambient temperature higher than 30°C will exhibit heat stress symptoms, resulting in reduced feed consumption, reduced feed utilization and feed efficiency, inhibition of viability and fecundity, and limited meat quality (Marai et al., 2002; Bakr et al., 2015; Camp et al., 2018). Therefore, a better understanding of the physiological changes of microbial components and metabolites under high temperature will help to develop special methods to deal with high temperature rabbit raising in the future. In the current study, 16SrRNA sequencing was combined with metabonomics to explore the intestinal microbial diversity and composition and the subsequent changes of fecal metabolites in HS rabbits.

Animal health was affected directly by HS, causing oxidative stress and immune suppression, leading to mortality (Lacetera, 2019). According to the current study, the serum IgG level in the HS group was higher than in the CON group. Immunoglobulin has been established in previous research to have a crucial function in immune regulation and mucosal defense. IgG is the highest serum immunoglobulin, which can reflect the systemic immune status of animals (Li et al., 2019). The findings suggest that HS may affect rabbits’ immune function to some extent. T-AOC is an important index to evaluate the antioxidant capacity (Wei et al., 2020). In the current experiment, the expression of T-AOC in the HS group was reduced, suggesting that rabbits’ antioxidant capacity had diminished and that oxidative stress may occur. TNF-α is a proinflammatory factor (Li et al., 2019), whereas IL-10 is a typical anti-inflammatory cytokine (Maynard et al., 2007). IL-10 is essential in conditioning intestinal homeostasis by inhibiting the release of inflammatory mediators, promoting natural and specific immunity (Maynard et al., 2007). IL-10 is most classically associated with Foxp3^+^ Tregs, and it inhibits lymphocyte and bone marrow immunity *via* several mechanisms (Castro-Dopico and Clatworthy, 2019). In the current experiment, the expression of IL-10 was downregulated, while TNF-α was upregulated in HS the group, indicating that HS may trigger an inflammatory response in rabbits.

We used metagenomic sequencing to investigate active fecal microbiota alterations in rabbits to relate the fecal microbiome to heat stress. Our findings suggest that *Firmicutes* and *Bacteriodota* are the two most prevalent phyla in rabbits, consistent with earlier research (Velasco-Galilea et al., 2018; Shanmuganandam et al., 2020). We also discovered taxonomic and functional changes between the CON and HS groups in the fecal microbiome. At the phylum level, our research results show that *Firmicutes* and *Bacteroidetes* are the two main groups, followed by *Proteobacteria*, *Verrucomiceobiota*, *Desulfobacterota*, *Chloroflexi*, *Coprothermobacterota*, *Euryarchaeota*, and *Actinobacteriota*, which is consistent with the research results of Jandhyala et al. (2015) and Velasco-Galilea et al. (2018). It should be noted that HS increased the relative abundance of *Firmicutes* by 11% while decreasing the relative abundance of *Bacteroides* by 7%. Traditionally, the *Firmicutes/Bacteriodota* ratio has been associated with susceptibility to disease status (Ley et al., 2006). Bin et al. (2018) showed that the ratio of *Firmicutes/Bacteriodota* increased in the jejunum of diarrhea piglets and determined that the increased ratio of *Firmicutes/Bacteriodota* was related to diarrhea. *Bacteroides* is an anaerobic Gram-negative bacterium that is found in the mammalian gastrointestinal microbiota (Jandhyala et al., 2015; Rodríguez et al., 2015). The genus is critical for vegetal polysaccharides breakdown and amino acid fermentation (Dai et al., 2011; Fang et al., 2017). *Firmicutes* are linked to obesity because they may ferment plant polysaccharides to create short-chain fatty acids (SCFA) to provide additional energy (Ley et al., 2006). These findings imply that raising the *Firmicutes/Bacteriodota* ratio may increase pathogen adhesion and colonization in the intestine. As a result, we hypothesize that rabbits exposed to HS will be more susceptible to a variety of diseases, have lower immunity, and suffer from diarrhea and other diseases and that the imbalance of intestinal flora caused by HS will change the fermentation and digestion mode of plants in rabbits, making rabbits more prone to pathological obesity.

In addition to variations in relative abundance at the phylum level, certain discrepancies at the genus taxonomic level were detected. HS boosted the abundance of *Akkermansia (Verrucomicrobiota)* in the current research. Current studies have elucidated that these species also contribute to the reparation of mucosal wounds (Alam et al., 2016), and they could be employed as probiotics (Gómez-Gallego et al., 2016). Furthermore, Borton et al. (2017) found that low levels of inflammation increased the relative number of these bacteria in the mouse stomach. It is worth noting that *Akkermansia* is positively correlated with imidazolectic acid, an antioxidant effect (Tansini et al., 2004). Therefore, we speculate that rabbits exposed to HS have a low immune function, anti-inflammatory response, and the destruction of intestinal mucosal tissue, whereas *Akkermansia* positively regulates the production of antioxidant metabolites, protects the self-healing of the intestinal mucosal protective layer, and enhances the response to inflammatory reaction damage. It demonstrates that HS causes an increase in unfavorable bacteria in the rabbit gut, reduces rabbit production, and may cause diarrhea and enteritis. *Clostridium sensu stricto 1 (Firmicutes)* was likewise shown to be considerably upregulated in the HS group. *Clostridium sensu stricto 1* is harmful to mammalian intestinal health. *Clostridium sensu stricto 1* expression was significantly upregulated in the colon of high food diet. It is speculated that the enrichment of *Clostridium sensu stricto 1* may cause the colonic epithelial inflammation of sheep (Wang et al., 2017). In the HS group, *Escherichia-Shigella (Proteobacteria)* was significantly downregulated, linked with a proinflammatory state (Soares et al., 2012; Morgan, 2013). *Escherichia-Shigella* persistent infection can result in chronic and persistent peripheral inflammation (Small et al., 2013). In addition, it can induce the production of proinflammatory cytokines (De la Fuente et al., 2014). The biological control system is dynamic and complicated. The reasons the relative abundance variances at the categorization level differ from the findings mentioned above are unclear and must be investigated further. These discrepancies may be due to species, feed, location, and environmental differences.

According to the functional prediction findings, HS might improve necrotic apoptosis, different amino acid metabolism, gluconeogenesis, glycolysis, glyoxylic acid, dicarboxylic acid metabolism, pyruvate metabolism, butyric acid metabolism, and the insulin signaling system. On the contrary, HS significantly reduced the metabolic pathways of inositol phosphate metabolism, glycospholipid metabolism, and the phosphotidylinositol signaling system, indicating that rabbit metabolism differed depending on temperature. The differential metabolites primarily focus on related endogenous chemicals, particularly in metabolic pathways such as tryptophan production, VB6 metabolism, and prolactin signaling *via* the cytochrome P450 metabolic pathway. Necroptosis is a newly defined type of regulated necrosis that plays a role in various inflammatory diseases. Ma et al. (2019) reported that HS increased necroptosis in broilers. Huang et al. (2020) showed that HS, through MAPK and NF, is related to cell growth, differentiation, migration, aging, inflammatory response, and apoptosis-κB, and C-Jun signaling pathways induce RIP1/RIP3 dependent necroptosis. Tryptophan *de novo* synthesized nicotinamide adenine dinucleotide (NAD^+^) *via* the canine urinary ammonia pathway (Castro-Portuguez and Sutphin, 2020). The kynurenine pathway’s activity influences NAD + control of ROS levels and mitochondrial function (Cantó et al., 2009). In turn, NAD^+^ regulates the TCA cycle and mitochondrial function, epigenetic landscape, DNA repair, and hypoxia response (Castro-Portuguez and Sutphin, 2020). Leukotriene B4 (LTB 4) is essential for innate immunity. Activating the appropriate G protein-coupled receptor attracts and activates neutrophils (GPCR) (Smith et al., 1984). Superoxide anion (O_2_^–^) released by neutrophils plays an essential role in the antibacterial host defense system and tissue autologous injury. Finally, HS promotes oxidative stress in rabbits, resulting in mitochondrial malfunction and DNA damage. Oxidative stress generates several chemical substances, including prostaglandins, serotonin, and leukotrienes, which attract neutrophils, and neutrophils then attract other leukocytes and lymphocytes by producing cytokines. Under HS, the immune system plays a role in boosting the body’s self-healing. IDO1, IDO2, and TDO2 mediate tryptophan entry into canine urine (Cervenka et al., 2017). *Alistipes* are mainly involved in histidine degradation, THF, indole, and phenol production (Kaur et al., 2017). Metabolite of kynurenine XA may impede insulin/IGF-1 signal transduction in islets, whereas high pyridoxine inhibits endogenous XA production, resulting in a drop in blood glucose. Methionine may activate GCN5 acetyltransferase and enhance transcription coactivator PGC-1α acetylation in the transamination pathway, influencing hepatic gluconeogenesis and modifying blood glucose levels (Tavares et al., 2016). Inositol phosphate may also change the role of second messenger molecules in the energy metabolism pathway of insulin-sensitive tissues (including liver, muscle, and adipose tissues) (Chatree et al., 2020). As a result, HS may alter the intestinal flora, which alters the way rabbits digest and ferment food, affecting the level of metabolites in the intestine. Differential metabolites are primarily involved in insulin inhibition, blood glucose levels, gluconeogenesis, and energy metabolism, which may be the primary causes of HS rabbits’ decrease of appetite, feed consumption, feed utilization, and feed efficiency (Sirotkin et al., 2021). In short, the harm caused by HS to the human body is a dynamic process that can fluctuate with time and intensity; the outcomes may change depending on the research settings. Furthermore, HS may operate as the initial messenger to regulate inositol molecule, insulin signal, amino acid metabolism, and glucose metabolism network and respond to and heal body damage (Tang et al., 2021; Wen et al., 2021).

In conclusion, the current study revealed the critical relationship between intestinal microbiota structure and metabolism under heat stress. Under HS conditions, the intestinal microbiota imbalance is closely related to intestinal dysfunction and serum oxidative stress markers. We must identify the changes of intestinal flora structure and understand the correlation between microbiota and disease under heat treatment. In our study, we screened some metabolites and microbiota in heat stressed rabbit feces, which may have potential beneficial or harmful properties. In the future, rabbit raising can be realized by increasing beneficial bacteria and related metabolites in feed and drinking water (Oladimeji et al., 2021). In conclusion, the current study provides a theoretical and experimental basis for further study of high temperature injury in humans and other animals. However, further functional analysis is needed to confirm whether the structural changes and metabolite differences of microbiota are related to intestinal dysfunction in rabbits.

## Data Availability Statement

The datasets presented in this study can be found in online repositories. The names of the repository/repositories and accession number(s) can be found below: NCBI, accession: PRJNA790744; BioSample ID: SAMN24224881-SAMN24224892 (https://www.ncbi.nlm.nih.gov/bioproject/?term=PRJNA790744).

## Ethics Statement

The animal study was reviewed and approved by all applicable international, national, and/or institutional guidelines for the care and use of animals were followed.

## Author Contributions

XB, YS, LT, HW, JW, SC, XJ, and SL participated in the experiment conception and design. XB, YS, LT, and LC carried out the experiment. XB analyzed the experimental data and drafted the manuscript. YS and HF revised the manuscript. All authors read and approved the manuscript.

## Conflict of Interest

The authors declare that the research was conducted in the absence of any commercial or financial relationships that could be construed as a potential conflict of interest.

## Publisher’s Note

All claims expressed in this article are solely those of the authors and do not necessarily represent those of their affiliated organizations, or those of the publisher, the editors and the reviewers. Any product that may be evaluated in this article, or claim that may be made by its manufacturer, is not guaranteed or endorsed by the publisher.

## Footnotes

1
http://ccb.jhu.edu/software/FLASH/

2
http://qiime.org/scripts/split_libraries_fastq.html

3
https://github.com/torognes/vsearch/

4
http://www.drive5.com/uparse/

5
http://www.drive5.com/muscle/

6
https://www.mzcloud.org/

7
http://www.r-project.org/
